# Mountain gorilla lymphocryptovirus has Epstein-Barr virus-like epidemiology and pathology in infants

**DOI:** 10.1038/s41598-017-04877-1

**Published:** 2017-07-13

**Authors:** Tierra Smiley Evans, Linda J. Lowenstine, Kirsten V. Gilardi, Peter A. Barry, Benard J. Ssebide, Jean Felix Kinani, Fred Nizeyimana, Jean Bosco Noheri, Michael R. Cranfield, Antoine Mudakikwa, Tracey Goldstein, Jonna A. K. Mazet, Christine Kreuder Johnson

**Affiliations:** 1 Karen C. Drayer Wildlife Health Center, One Health Institute, School of Veterinary Medicine, University of California, Davis, CA 95616 USA; 2 Department of Pathology, Microbiology and Immunology, School of Veterinary Medicine, University of California, Davis, CA 95616 USA; 3 Center for Comparative Medicine, Department of Pathology and Laboratory Medicine, California National Primate Research Center, University of California, Davis, CA 95616 USA; 4 Gorilla Doctors, Mountain Gorilla Veterinary Project, Inc., Kampala, Uganda; 5 One Health Approach for Conservation, Gorilla Health, Kigali, Rwanda; 6 Gorilla Doctors, Mountain Gorilla Veterinary Project, Inc., Musanze, Rwanda; 7 Rwanda Development Board, Kigali, Rwanda

## Abstract

Epstein-Barr virus (EBV) infects greater than 90% of humans, is recognized as a significant comorbidity with HIV/AIDS, and is an etiologic agent for some human cancers. The critically endangered mountain gorilla population was suspected of infection with an EBV-like virus based on serology and infant histopathology similar to pulmonary reactive lymphoid hyperplasia (PRLH), a condition associated with EBV in HIV-infected children. To further examine the presence of EBV or an EBV-like virus in mountain gorillas, we conducted the first population-wide survey of oral samples for an EBV-like virus in a nonhuman great ape. We discovered that mountain gorillas are widely infected (n = 143/332) with a specific strain of lymphocryptovirus 1 (GbbLCV-1). Fifty-two percent of infant mountain gorillas were orally shedding GbbLCV-1, suggesting primary infection during this stage of life, similar to what is seen in humans in less developed countries. We then identified GbbLCV-1 in post-mortem infant lung tissues demonstrating histopathological lesions consistent with PRLH, suggesting primary infection with GbbLCV-1 is associated with PRLH in infants. Together, our findings demonstrate that mountain gorilla’s infection with GbbLCV-1 could provide valuable information for human disease in a natural great ape setting and have potential conservation implications in this critically endangered species.

## Introduction

Epstein-Barr virus (EBV), a gamma herpesvirus in the *Lymphocryptovirus* genus, is one of the most widespread and prevalent human pathogens, infecting over 90% of humans and persisting for their lifetimes^1^. In contrast with more developed countries, where people are primarily infected during adolescence, people living in less developed countries are usually first infected as infants^2, 3^. In infants, primary infection is either asymptomatic or associated with non-specific signs of a viral infection^4^. The prevalence of histolopathologic changes associated with primary infections in infancy is largely unknown because tissue biopsies are rarely collected. However, EBV has been implicated in the development of a wide variety of benign and malignant diseases associated with congenital or acquired immune dysfunction and has become of increasing concern with the prevalence of HIV/AIDS globally. In young and adult humans with immune dysfunction, EBV has been associated with pulmonary reactive lymphoid hyperplasia, endemic Burkitt’s lymphoma, undifferentiated nasopharyngeal carcinoma, and extra-nodal B-cell lymphomas^5–7^.

In nonhuman primates (hereafter referred to as primates), EBV-like lymphocryptoviruses have been associated with lymphoproliferative disorders and malignancies similar to those reported in immunodeficient humans^8^. In captive primates, lymphocryptoviruses have been associated with B-cell nasal lymphoma in simian immunodeficiency virus (SIV)-infected rhesus macaques, non-Hodgkin lymphomas in SIV-infected rhesus and cynomolgus macaques, and T-cell lymphomas in SIV-infected pigtail and otherwise healthy Japanese macaques^9–17^. EBV-like lymphocryptoviruses are known to infect great apes: two types of lymphocryptoviruses similar to type 1 and type 2 EBV in humans have been detected in western lowland gorillas (*Gorilla gorilla gorilla*; GgorLCV1 and GgorLCV2) and orangutans (*Pongo* spp; PpygLCV-1 and PpygLCV-2), and a lymphocryptovirus similar to type 1 EBV has been detected in chimpanzees (PtroLCV-1)^18, 19^. Because it is difficult to conduct population-wide studies to detect viruses circulating in wild great apes, little is known about the epidemiology or associated pathologies of EBV-like infections in great apes, particularly those associated with primary infection in infancy.

Mountain gorillas (*Gorilla beringei beringei*) are at high risk for contracting human pathogens because approximately 60% of their population is habituated to the presence of humans^20, 21^. Habituation involves gradually familiarizing selected gorilla groups to the close presence of humans to ensure gorillas are accessible for conservation-related management, research, and tourism^22^. While habituation is a highly effective conservation strategy, it can expose great apes to pathogens^23^. Herpesviruses are chronically shed in saliva (between 84 and 90% of human adults have been shown to orally shed EBV^24, 25^) and can remain stable in the environment for up to one week providing opportunities for mountain gorillas to contact viruses that humans have shed in parks or captive enclosures^26^. The potential for close proximity contact in captivity has been demonstrated through the detection of human herpes simplex virus-1 (HSV-1), an alphaherpesvirus, in a confiscated eastern lowland gorilla (*Gorilla beringei graueri*) cared for by human caretakers using a similar management strategy to confiscated mountain gorillas^27^. Wild habituated mountain gorillas are also frequently documented to be in close proximity with humans^28^. While gammaherpesviruses are generally considered to be the most host specific of the herpesvirus subfamilies, a population level survey of potential cross-species herpesviral infections in great apes has not yet been performed. In experimental infections, EBV has the ability to infect multiple species including cotton-top tamarins, owl monkeys, common marmosets, rhesus macaques and cynomolgus monkeys^29–33^. In addition, a recent analysis of gammaherpesviral sequences from a wide range of mammals indicated that cross-species transmissions have historically occurred more frequently than previously estimated, with most attributable to bats and primates^34^.

In previous studies, all wild mountain gorillas sampled and tested were serologically positive for antibodies to human EBV^35^. However, it was unclear if these serological positives represented exposure to human EBV or cross-reactivity of the assay with a related virus. In addition, pulmonary reactive lymphoid hyperplasia (PRLH), consisting of lymphocytic interstitial pneumonia and/or follicular bronchiolitis^7^, a syndrome associated with primary EBV infection in immunosuppressed human infants and children^36, 37^, has been seen histologically in lung tissues collected post-mortem from mountain gorilla infants.

Here we investigated the epidemiology and origin of lymphocryptoviruses shed by wild human-habituated mountain gorillas in the two remaining mountain gorilla subpopulations, the Virunga gorillas residing in the Virunga Massif (spanning Rwanda, Uganda, and the Democratic Republic of Congo) and the Bwindi gorillas residing in the Bwindi Impenetrable Forest in Uganda. To date, investigation of great ape populations for orally shed viruses has been limited by the ability to non-invasively collect oral samples^38^. We implemented a novel chewed plant method to collect oral samples at a population-wide scale, for the first time in a great ape. We also conducted a retrospective study of infant necropsy cases over a 10-year period to examine mountain gorillas for naturally occurring asymptomatic primary EBV-like lesions. Our findings demonstrate that mountain gorillas are widely infected with a mountain gorilla-specific lymphocryptovirus with epidemiologic and pathologic similarities to EBV in humans.

## Results

### Identification of lymphocryptovirus oral shedding in wild Bwindi and Virunga mountain gorillas

**Figure 1:**
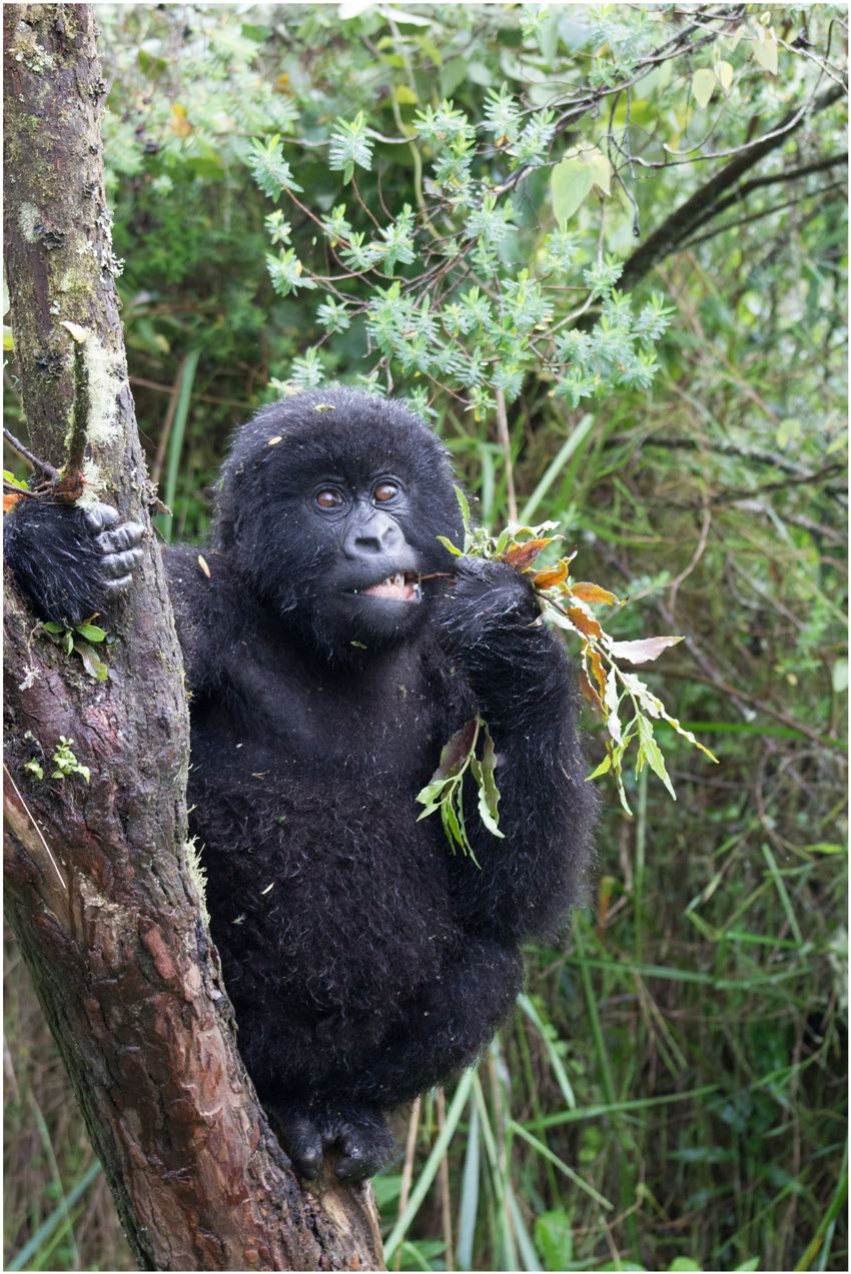
Infant Virunga mountain gorilla chewing and discarding plant pieces in the Volcanoes National Park, Rwanda.

To study oral shedding of lymphocryptoviruses, discarded chewed plants were collected from 76.2% of the Bwindi and 47.6% of the Virunga human-habituated mountain gorillas (n = 294 gorillas from 26 family groups) (Fig. 1). These two mountain gorilla populations are estimated to have been genetically isolated from each other for more than 5,000 years^39^. Using consensus polymerase chain reaction (PCR) we applied broadly reactive degenerative primers to characterize the herpesviral DNA present on chewed plants targeting the DNA polymerase (DPOL), terminase (TERM) and glycoprotein B (gB) herpesviral genes. We identified the first evidence of an EBV-like virus, a mountain gorilla-specific strain of lymphocryptovirus 1 (GbbLCV-1), in wild mountain gorillas as well as herpesviruses in the cytomegalovirus and rhadinovirus subfamilies. Amplified lymphocryptovirus sequences did not show significant differences between Bwindi and Virunga gorillas, with 98.9% nucleotide and 98.7% amino acid similarity across a 473 bp fragment of the gB gene (Genbank accession #’s KU736789, KU736790); 99.6% nucleotide and 100% amino acid similarity across a 230 bp fragment of the DPOL gene (Genbank accession #’s KU736786, KU736788); and 98.8% nucleotide and 97.9% amino acid similarity across a 424 bp fragment of the TERM gene (Genbank accession #’s KU736787, KU736791). Analysis of the gB gene fragments showed 97.7–97.9% nucleotide and 99.4% amino acid similarity to GgorLCV-1 isolated from western lowland gorillas (AF534225) and 91.3–91.5% nucleotide and 96.2–97.5% amino acid similarity to human EBV (LN827799). Analysis of the DPOL fragment showed 98.9–99.3% nucleotide and 98.7–100.0% amino acid similarity to GgorLCV-1 (KU578068) and 89.7–90.2% nucleotide and 91.9–93.2% amino acid similarity to EBV (AB850649). TERM sequences for GgorLCV-1 were not available in the Genbank database. Sequences detected in all mountain gorillas were most similar to GgorLCV-1 and did not show a close similarity to GgorLCV-2 (75.9–76.5% nucleotide and 74.5–75.8% amino acid similarity to GgorLCV-2 for the gB gene fragment; AY129395). Finding that GbbLCV-1 is most closely related to the western lowland gorilla lymphocryptovirus-1 is to be expected given the close phylogenetic relatedness of the host species.

### Geographic distribution, demographic associations and prevalence of GbbLCV-1 in wild mountain gorillas

Mountain gorillas were identified by name, age, sex and family group at the time of oral sample collection in order to investigate potential associations between demographic factors and lymphocryptovirus oral shedding. In addition, archived mountain gorilla blood samples, which had been collected during opportunistic interventions, were examined to identify lymphocryptoviruses circulating in white blood cells (n = 23). We found oral shedding of GbbLCV-1 in 43.0% (143/332) of mountain gorillas, with 48.8% (83/170) of gorillas in the Virunga Massif and 37.0% (60/162) of gorillas in the Bwindi Impenetrable Forest orally shedding GbbLCV-1. Our estimates are likely lower than the true prevalence, given the rugged field conditions surrounding oral specimen collection from chewed plants and the likelihood of intermittent shedding^40^. Prevalence of GbbLCV-1 was not significantly different among conservation regions, age, sex, or family group (Supplementary Table 1).

The mountain gorilla-specific strain of LCV-1 was also detected in circulating white blood cells in 11 of 15 adult females, 1 of 2 adult males, 2 of 4 juvenile females, 0 of 1 juvenile males, and 1 of 1 infant females from whom blood samples were opportunistically collected. No significant differences were detected between age or sex and lymphocryptovirus presence in circulating white blood cells. While the availability of opportunistically collected blood samples was low, the high prevalence of GbbLCV-1 in gorilla oral secretions tested from the majority of the habituated population and frequent detection in gorilla peripheral white blood cell samples strongly suggest widespread infection with GbbLCV-1 in both mountain gorilla subpopulations.

### Patterns of GbbLCV-1 transmission in wild mountain gorillas

To determine under what circumstances mountain gorillas acquire GbbLCV-1 in the wild, we screened mountain gorillas from all age groups in which social structure was well known. Mountain gorillas from the 26 family groups sampled are followed daily and their reproductive states and social interactions are recorded. Thus, for the majority of mountain gorillas in these groups, their exact date of birth is known. The majority of mountain gorillas shedding GbbLCV-1 were infants (samples collected from infants aged 6 months to 3 years of age), suggesting primary infection during this stage of life, similar to what is seen in humans in less developed countries^41^. Fifty-two percent of infant mountain gorillas tested were orally shedding GbbLCV-1 (9/19 Bwindi and 16/29 Virunga gorilla infants; Table 1). All live infants sampled in this study appeared to be healthy and thriving, and no morbidity or mortality over a one-year post-sampling period was reported indicating that primary infection with GbbLCV-1 in healthy infants is asymptomatic or causes mild symptoms, similar to what is documented in human infection during infancy^4^. In addition, mother and infant pairs tended to be strongly correlated in shedding status. Specifically, ten mother-infant pairs were both shedding GbbLCV-1 in saliva and six mother-infant pairs were both not shedding at the time of sampling, while two mother-infant pairs had only the mother shedding and one mother-infant pair had only the infant shedding. Infant GbbLCV-1 shedding status was not significantly different from that of their mother’s (McNemar’s P value = 0.5) indicating mothers are most likely the primary source of infection for their infants. Mountain gorilla mothers were frequently observed grooming their infants, which could expose them to saliva containing lymphocryptovirus. In addition, infants, while still primarily nursing, were frequently observed investigating and chewing on discarded chewed plants that their mothers had dropped. Samples chewed on by both mother and infant were not collected for diagnostic analyses.

**Table 1:** Mountain gorillas (*Gorilla beringei beringei*) from the Virunga Massif and Bwindi Impenetrable Forest shedding mountain gorilla-specific lymphocryptovirus-1 (GbbLCV-1) between November 2012 and June 2013 by age class.

Age Group*	#positive/#tested	Period Prevalence	95% CI^†^
Infant	25/48	0.52	0.37–0.67
Juvenile	22/59	0.37	0.25–0.50
Subadult	3/13	0.23	0.05–0.54
Adult	89/201	0.44	0.37–0.51

^*^Infants were defined as gorillas less than 3 years of age, juveniles as 3 to 5 years of age, subadults as 6 to 7 years of age and adults as 8 years and older^64, 65^. ^†^CI, 95% binomial exact confidence intervals^66^.

### Pulmonary reactive lymphoid hyperplasia (PRLH) cases

**Figure 2:**
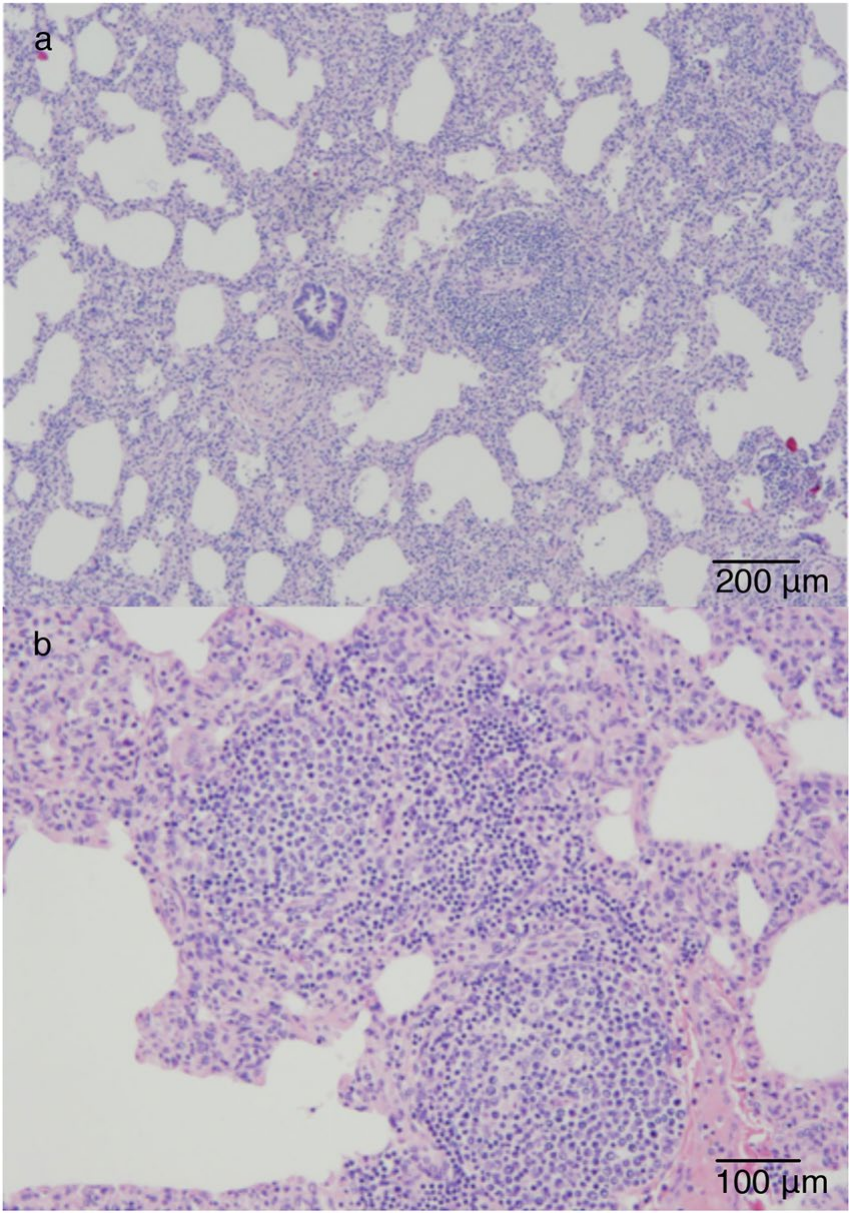
Pulmonary reactive lymphoid hyperplasia in an infant mountain gorilla (*Gorilla beringei beringei*). Histological lesions included lymphocytic interstitial pneumonia exemplified by diffuse infiltration of the alveolar septa by lymphocytes (a and b).

To study the role of lymphocryptoviruses in the development of PRLH in mountain gorillas, we examined archived tissue samples from infants demonstrating histologic evidence of PRHL at death. Pulmonary reactive lymphoid hyperplasia has been identified in 11 out of 62 mountain gorilla infants (17.7%) that died between 1988 and 2013. We examined available post-mortem tissues from eight infants that died between 2001 and 2011, which demonstrated histological signs of PRLH. Histological lesions included lymphocytic interstitial pneumonia exemplified by diffuse infiltration of the alveolar septa by lymphocytes (Fig. 2) and follicular bronchiolitis exemplified by hyperplasia of the bronchial associated lymphoid tissue. In all of the infants examined by necropsy in this study, PRLH was incidental to other unrelated primary causes of death. One infant had a congenital cleft palate, one was killed due to infanticide, and six died as a result of maternal abandonment and exposure. Respiratory signs were not identified in any study infants prior to death despite respiratory illness being well-documented in the mountain gorilla population^23^. Evidence of immune dysregulation was present in the form of lymphadenopathy characterized by giant follicular hyperplasia in two of these cases and thymic atrophy in two.

### Localization of lymphocryptoviruses in infant lung tissues

To test the association of PRLH with primary lymphocryptovirus infection in gorillas, we compared GbbLCV-1 DNA presence/absence in available frozen post-mortem tissues from eight infants with histologic evidence of PRLH at death, three infants with no evidence of PRLH, and one adult female with no evidence of PRLH at death (Table 2). The mountain gorilla-specific strain of lymphocryptovirus -1 was detected in at least one available tissue type from 5 of 8 (62.5%) gorillas with histologic evidence of PRLH, including 5 of 7 (71.4%) lung tissue samples with corresponding lung lesions (frozen lung tissue was not available from one PRLH pathology case; Table 2). We did not detect GbbLCV-1 in any lung tissue from gorillas without histologic evidence of PRLH (Table 2). Our findings suggest an association between GbbLCV-1 infection and development of PRLH in mountain gorillas, similar to the development of EBV-related PRLH in humans.

**Table 2:** Mountain gorilla-specific lymphocryptovirus 1 (GbbLCV-1) infection and lesions detected in post-mortem tissue samples from wild infant mountain gorillas (*Gorilla beringei beringei*) in the Virunga Massif with and without pulmonary reactive lymphoid hyperplasia.

Gorilla*	Pathology Present	Tissue Tested	Lesions in Tissues Tested	PCR results for GbbLCV-1
**Suspected EBV-like Infant Pathology Cases**				
1	LIP, FB, GFH**	liver	NSL	−
		lung	LIP, FB	−
		kidney	NSL	−
		spleen	NSL	−
2	LIP	liver	Lipidosis	−
		R. kidney	NSL	−
		L. kidney	NSL	−
		spleen	NSL	−
		R. lung	LIP	−
		L. lung	LIP	−
3	LIP and FB	kidney	NSL	−
		spleen	NSL	+
		lung	LIP	+
4	LIP and FB	kidney	NSL	−
		spleen	RH	+
		liver	Atrophy	−
		lung	LIP	+
		pancreas	ZD	−
5	LIP	lung	LIP	+
		spleen	NSL	−
		liver	CP	+
		kidney	NSL	−
6	LIP and GFH	Lung	LIP	+
7	LIP	kidney	NSL	−
		liver	NSL	−
		lung	LIP	+
8	LIP	blood clot	NSL	−
**Non**−**suspected EBV**−**like Pathology Cases**				
9	Not suspected	L. Lung	BP	−
		R. Lung	BP	−
10	Not suspected	spleen	NSL	−
		liver	NSL	−
11	Not suspected	liver	NSL	−
		tongue	Possible inclusions	−
		esophagus	EP	−
12	Not suspected	kidney	Mild nephritis	−
		liver	NSL	−
		lung	NSL	−
		spleen	NSL	+
		mouth lesion	Stomatitis	−

*Gorilla #1 = Bukima’s infant, #2 = Tayina’s infant, #3 = Bishushwe’s infant, #4 = Iradua, #5 = un-named infant, #6 = Mutesi, #7 = Faida’s infant, #8 = Icyi, #9 = Tweshimwe’s infant, #10 = Nzeli’s infant, #11 = Amatwara, #12 = Tuck; **LIP = Lymphocytic interstitial pneumonia, FB = follicular bronchiolitis, NSL = no specific lesions, RH = reactive hyperplasia, BP = bronchopneumonia, EP = esophagitis, CP = capillariasis, ZD = zymogen depletion.

### Risk of cross-species transmission of EBV to Eastern gorillas

To further evaluate whether extremely close human-gorilla contact could lead to cross-species infection with human EBV, in addition to our screening of the wild mountain gorilla population (described above), we tested direct oral swab samples from one confiscated mountain gorilla and five confiscated eastern lowland gorillas being reared in captivity in very close contact with human caregivers. We used the same pan-herpesvirus PCR assays, designed to detect herpesviruses in the alpha, beta and gamma subfamilies and the EBV specific PCR assay, for both wild and confiscated gorillas. The potential for cross species transmission of viruses by close proximity contact has been previously demonstrated with the detection of HSV-1 in one of the captive eastern lowland gorillas in this study^28^. All six confiscated gorillas were orally shedding GbbLCV-1 at the time of sample collection. Gorilla orphans were brought into captivity between the approximate ages of 6 months and 3 years and were likely infected with GbbLCV-1 prior to being removed from the wild. Human EBV was not detected in these captive gorillas or in any wild gorillas tested in this study using our methods.

## Discussion

Contrary to previous serologic evidence for ubiquitous EBV exposure in mountain gorillas^35^, our population-wide survey did not detect shedding of EBV in the wild or captivity during our study period and alternatively demonstrated that the lymphocryptovirus circulating in mountain gorillas is a mountain gorilla-specific strain of lymphocryptovirus 1, GbbLCV-1. Epstein-Barr virus serologic assays used previously were likely cross-reactive with GbbLCV-1. We also identified multiple epidemiologic and pathologic similarities between GbbLCV-1 and EBV including: primary infection during infancy, a likely mother-to-infant transmission pathway via saliva, latent infection in peripheral white blood cells, intermittent oral shedding and an association between primary infection and the presence of PRLH. Together these similar epidemiologic and pathologic findings suggest that GbbLCV-1 could be used to gain insight into human disease. While experimental manipulation of mountain gorillas would not be feasible or ethical in the wild, information can be gained from noninvasive health studies and pathological investigations of deceased individuals, providing valuable information for human disease in a natural great ape setting.

Mountain gorilla infants had the highest prevalence of GbbLCV-1 oral shedding compared to other age groups. It is likely that infants are protected by maternal antibodies until approximately 6 months of age but become infected shortly thereafter when maternal antibodies wane, similar to what has been documented for lymphocryptoviruses in rhesus macaques and in humans in less developed countries^13, 41^. Oral shedding of GbbLCV-1 was not detected in any infant under the age of 6 months. In less developed countries, human primary infection with EBV typically occurs during infancy through exposure to saliva by close contact with their mothers (in part due to the practice of pre-mastication of food for infants) or other family members and is either asymptomatic or results in nonspecific signs of a viral infection^2, 3, 42, 43^. In developed countries, human primary infection with EBV typically occurs during adolescence when people begin to be exposed to saliva through contact with other adolescents (thus it’s common name as the “*kissing disease*”), and infection in this age class can cause mononucleosis^44^. In this study, we identified a correlation between mother and infant GbbLCV-1 oral shedding, which suggests mothers pass the virus to their infants through infected saliva. Infant mountain gorillas frequently explore and chew on discarded food items from their mothers and mother-infant grooming is common; both activities which present opportunities for exposure to GbbLCV-1 through saliva.

While the highest prevalence of GbbLCV-1 oral shedding was in infants, lymphocryptovirus was detected in all age groups in both oral specimens as well as peripheral white blood cells. We know that after initial infection with EBV, the virus remains latent in peripherally infected B lymphocytes^45^. Detecting GbbLCV-1 in both oral specimens and peripheral white blood cells in multiple age groups suggests a similar route of infection and persistence in mountain gorillas.

Our study localized GbbLCV-1 viral DNA to five infant lung tissues demonstrating lesions consistent with PRLH. A lymphocryptovirus could not be detected in any negative control mountain gorilla lung tissues. Epstein-Barr virus DNA has been associated with PRLH lesions *in situ* in human case-control studies^46^. Although our sample size of mountain gorilla tissues viable for molecular diagnostics was small, identifying GbbLCV-1 in mountain gorilla PRLH lesions together with the knowledge that EBV is associated with PRLH in humans suggests a similar pathogenesis in mountain gorillas. None of the mountain gorilla infants in which PRLH lesions were identified showed signs of respiratory illness prior to their death, indicating that PRLH can occur without overt clinical signs.

While primate models for human infection with EBV exist in captivity^47^ and other great ape-specific lymphocryptoviruses have been identified in wild chimpanzees, western lowland gorillas and orangutans, mountain gorillas present a more accessible model for lymphocryptovirus epidemiology and pathology in a natural free-ranging host. The majority of individuals in the mountain gorilla population are identified by name with researchers and park personnel monitoring them daily. More than four decades of data on life histories and behavioral interactions are recorded for these human habituated gorillas^48^. Until recently, population-wide studies of orally shed viruses in mountain gorillas were logistically impossible, limiting virus data to that which could be obtained from fecal samples or infrequently collected blood or oral swab samples. With the introduction of chewed plants as a way to collect great ape oral samples, mountain gorillas can now be frequently monitored for oral shedding of lymphocryptoviruses non-invasively with minimal behavioral disruption^38^.

In addition, post-mortem infant mountain gorilla tissue samples are opportunistically available, permitting investigation of histopathologic lesions associated with primary lymphocryptovirus infection. Infant mountain gorilla mortality is high due to the practice of infanticide by adult males and other traumatic and infectious causes^49, 50^. When infants die in the forest, every attempt is made to collect their bodies for necropsy. While only a small number of appropriately preserved tissue samples from these necropsies were available for our retrospective study due to inconsistencies in sample collection and variable length of formalin fixation, investigations into infant mortality are ongoing and additional PRLH cases are being identified and investigated.

Paired molecular and histopathologic findings in gorilla infants, in conjunction with the overall high prevalence of PRLH in mountain gorilla infant necropsy cases, indicates that histopathologic lesions associated with primary infection with a lymphocryptovirus is common in mountain gorilla infants. In contrast, little is known about the histopathologic lesions associated with primary infection with EBV in human infants and children for whom invasive lung biopsies are rarely performed. In children, in whom PRLH was diagnosed with an open lung biopsy, it is usually associated with congenital or acquired immune dysregulation (*e*.*g*., Down syndrome, autoimmune diseases, and HIV/AIDS)^7^. Inferring from findings reported here in mountain gorillas, we hypothesize that pathology in humans is likely more common than has been reported to date and is more likely to be present in the absence of overt clinical signs. Our data warrant further investigation of the frequency of histopathologic lesions associated with primary infection with EBV in human infancy.

Among the lymphocryptoviruses detected in mountain gorillas, only sequences similar to GbbLCV-1 were detected in this study. Mountain gorillas were either not infected with a mountain gorilla-specific strain of lymphocryptovirus-2, or this virus was not detected using our methods. Previous studies have shown that the majority of western lowland gorillas infected with GgorLCV-2 were also co-infected with GgorLCV-1^18^. In cases of multiple herpesviral infections, degenerate herpesvirus assays can sometimes favor detection of one herpesvirus over another^51^. In humans, type 1 EBV is the most prevalent worldwide but type 2 is common in parts of Africa^52^. Type 1 transforms human B cells into lymphoblastoid cell lines much more efficiently than type 2 EBV^53^. Further studies are needed to investigate the presence of a type 2 EBV-like virus in the mountain gorilla population. B-cell lymphoma, a cancer that has been associated with EBV infection in humans, has been noted in one adult female mountain gorilla and GbbLCV-1 was detected by PCR in a blood clot from this gorilla collected at necropsy. Further investigation into naturally occurring cancers in mountain gorillas that may be associated with GbbLCV is warranted.

Investigation of herpesviruses can also be used to study primate population structure and migration patterns^54^. Phylogenetic analysis shows that herpesvirus divergence closely parallels mammalian, and in particular primate, speciation^55^. Interestingly there were very few nucleotide or amino acid sequence differences detected between the geographically isolated Bwindi and Virunga mountain gorilla populations. The Bwindi gorillas are an allopatric population living a minimum of 25 kilometers from the Virunga gorillas and have certain morphological and ecological differences but are considered mountain gorillas^56, 57^. Finding that GbbLCV-1 sequences are not significantly different between these two isolated populations is further evidence for their relatively recent geographic separation estimated at approximately 5,000 years ago^39^.

Identifying GbbLCV-1 has important implications for conservation management of the species. Although the sample size of captive gorillas for EBV detection was low, the absence of detection of EBV in this population, which is in very close contact with humans, as well as the absence of detection of EBV in the wild population, which also has contact with humans, suggest that the likelihood of future transmission of EBV to mountain gorillas is low. While mountain gorillas are exposed to human EBV in captive as well as in free-ranging settings, broad infection with their own endemic lymphocryptovirus may provide cross-protective immunity against infection with human EBV. Many EBV infection trials in nonhuman primates have failed and this may be due to cross-protective immunity with EBV related gamma-1-herpesviruses^58^. Cross-species transmission of gamma-herpesviruses between Old World primates and apes is also thought to be a rare event based on a study of predator-prey relationships^59^. In addition, it has been demonstrated that rhesus macaques cannot be infected with EBV because of unknown host restriction factors^60^. Exposure to and infection with EBV therefore at this time does not seem to pose a great risk to mountain gorillas in captivity or in the wild.

In conclusion, we provide the first molecular data on the widespread infection of both subpopulations of the mountain gorilla with an EBV-like lymphocryptovirus. We also identified an association between PRLH and GbbLCV-1 infection in infant mountain gorillas. Mountain gorilla infants should continue to be monitored for PRLH cases as well as older age groups for lymphoproliferative disorders that are known to be associated with EBV in humans. Mountain gorillas are a unique population, genetically similar to humans and closely monitored by researchers, which could be used to examine future patterns of EBV-related oncogenesis in a natural great ape setting.

## Materials and Methods

### Ethics statement

The study was approved by the Uganda Wildlife Authority, the Rwanda Development Board, and the Institutional Animal Care and Use Committee of the University of California, Davis (IACUC #17504, Public Health Service Animal Assurance #A3433–01, United States Department of Agriculture Registration #93-R-0433), and the study was conducted in accordance with the relevant guidelines and regulations.

### Mountain gorilla sample and data collection

Samples were collected from mountain gorillas in the Volcanoes National Park in Rwanda and the Mgahinga and Bwindi Impenetrable Forest National Parks in Uganda. Chewed plant samples (n = 383) discarded by wild human-habituated mountain gorillas were collected from 294 individual mountain gorillas between November 2012 and June 2013, as described previously^38^.

Whole blood samples were opportunistically collected from human-habituated mountain gorillas between October 1997 and July 2008 (n = 15 adult females, n = 2 adult males, n = 4 juvenile females, n = 1 subadult male, and n = 1 female infant) while they were anesthetized for treatment of a presumed human-induced illness or injury. Tissue samples examined in this study were collected during necropsies of mountain gorillas that died in the Volcanoes National Park between 2006 and 2011 (n = 65 tissue samples from 14 individuals). Oral swab samples were collected and placed in viral transport media (Remel M6™, Thermo Scientific, Lenexa, KS, USA) from one orphan mountain gorilla and five eastern lowland gorillas in captivity in Rwanda while they were anesthetized for their annual routine health examinations between December 2009 and November 2010. All samples were frozen and stored at −80 °C. All samples were imported to the University of California Davis under CITES permit # 13US117181/9.

### Sample processing

Chewed plant samples were processed according to previously described techniques^38^. DNA was extracted from whole blood samples using DNEasy® blood and tissue kits (Qiagen, Valencia, CA, USA). Total nucleic acid was extracted from approximately 100 ug of frozen tissue. Tissue samples collected at necropsy were cut into small pieces with scissors disinfected with 10% sodium hypochlorite after handling each sample, to prevent DNA cross-contamination between tissues and between animals. Total nucleic acid was extracted using the NucliSENS® MiniMAG® system (bioMérieux, Inc.). DNA and total nucleic acid were stored at −80 °Celsius.

### Molecular Diagnostics

DNA was analyzed by polymerase chain reaction (PCR) to detect herpesviral DNA utilizing degenerative primers amplifying a 450 bp region of the TERM gene and 225 bp region of the DPOL gene. Primers for the TERM and DPOL assays were described previously^61, 62^. Primers used were designed to amplify all herpesviruses within the alpha, beta and gamma herpesvirinae subfamilies and were tested in our laboratory (unpublished data) as well as in previous studies to determine their ability to detect human herpesviruses, including EBV^63^. Purified sample DNA (2 ul) or products of first-round PCR reactions (1 ul) were used as templates in PCR reactions with 25 ul reaction mixture containing 1 μM of each PCR primer, 200 μM of each deoxynucleotide triphosphate, 0.75 units DNA Polymerase AmpliTaq Gold (ThermoFisher Scientific, Waltham, MA, USA) and 5% DMSO (Signma Aldrich, St. Louis, MO, USA). For the TERM assay, in first and second round PCR, the reactions were kept at 95 °C for 3 min and then cycled 55 times with 20 sec of denaturation at 95 °C, 30 sec of annealing at 48 °C and 30 sec of strand extension at 72 °C, followed by a final extension step at 72 °C for 10 min. For the DPOL assay the conditions were the same except that the annealing temperature was reduced to 46 °C. In order to confirm the absence of EBV, samples testing positive for a gorilla herpesvirus by degenerative herpes PCR assays were also screened using an EBV specific PCR assay using previously described primers for a fragment of the EBV DPOL gene^64^. PCR reactions were the same as described for degenerative assays with the exception of omitting DMSO. Reactions were kept at 95 °C for 3 min and then cycled 50 times with 20 sec of denaturation at 95 °C, 30 sec of annealing at 62 °C and 30 sec of strand extension at 72 °C, followed by a final extension step at 72 °C for 10 min. In addition, a representative subset of samples from gorillas from both the Virunga Massif and Bwindi Impenetrable Forest were analyzed by PCR to detect a fragment of the gB gene (509 bp) to generate larger sequences for phylogenetic analysis. Primers for the gB gene were described previously^18^ and PCR conditions were the same as described for the DPOL assay. Parallel reactions were run for all samples amplifying a fragment of the mammalian beta-actin gene to test sample quality. PCR products of appropriate size were cloned using TOPO® TA cloning kits (Invitrogen, Carlsbad, CA, USA), and sequencing was performed using Sanger sequencing at the University of California, Davis DNA sequencing laboratory. Sequences were compared to other published herpesviral sequences in the GenBank Database (National Center for Biotechnology Information, National Library of Medicine, Bethesda, Maryland, USA). Sequences were edited using Geneious version 7.0.6. Alignments were constructed using CLUSTAL W, executed through Geneious.

### Histological Diagnostics

Tissues collected at necropsy were fixed in 10% neutral buffered formalin. Fixation time was prolonged, up to more than a year in some cases, pending receipt of permits for importation into the United States. Fixed tissues were processed routinely for wax embedment and sectioning at 5 microns. Sections were stained with hematoxylin and eosin (H&E) and examined by light microscopy by a board certified veterinary pathologist (LJL).

### Statistical analysis

Wild mountain gorillas were divided into age classes: infants were defined as gorillas less than three years of age, juveniles as three to five years, subadults as six to seven years and adults as eight years and older^65, 66^. A total of 332 samples (out of 383) were included in statistical analyses (11 samples could not be properly assigned to an age group); samples that tested negative for herpesviruses but were also negative for mammalian beta-actin were excluded from statistical calculations because inadequate collection of quality sample material could not be ruled out for these samples. Period prevalence and 95% binomial exact confidence intervals (CI)^67^ for oral shedding of lymphocryptovirus were calculated across age class. Finite population correction factor was not used because sampling was conducted with replacement. Evaluation of the effects of conservation area, family group, age, and sex on gorilla lymphocryptovirus oral shedding was performed by both bivariate analysis using a 2-sided chi-square test and a mixed-effects logistic regression model^68, 69^. Associations between lymphocryptovirus detected in whole blood and potential risk factors, such as age and sex, as well as associations between lymphocytic interstitial pneumonia status and lymphocryptovirus detected in lung and non-lung tissues were evaluated using a Fisher’s exact test^70^. Association between oral lymphocryptovirus shedding in infants and their mothers was evaluated by using a McNemar’s test^71^. All statistical analyses were performed using STATA version 13.1^72^.

### Data Availability

The datasets generated during the current study are available from the corresponding author on reasonable request with permission from the Rwanda Development Board.

## Electronic supplementary material

Supplementary Figure 1
