# Supplementary material for: Mountain gorilla lymphocryptovirus has Epstein-Barr virus-like epidemiology and pathology in infants
Source: Sci Rep. 2017 Jul 13;7:5352. doi: 10.1038/s41598-017-04877-1 (PMC5509654; doi:10.1038/s41598-017-04877-1)
Supplement: Supplementary file 1 — Supplementary Figure 1 [file 41598_2017_4877_MOESM1_ESM.pdf]

Mountain gorilla lymphocryptovirus has Epstein-Barr virus-like epidemiology and pathology in infants

Tierra Smiley Evans, Linda J. Lowenstine, Kirsten V. Gilardi, Peter A. Barry, Benard J. Ssebide, Jean Felix Kinani, Fred Nizeyimana, Jean Bosco Noheri, Michael R. Cranfield, Antoine Mudikikwa, Tracey Goldstein, Jonna AK Mazet, Christine Kreuder Johnson

Supplementary Table 1. Mountain gorillas (*Gorilla beringei beringei*) from the Virunga Volcanoes Conservation Region and Bwindi Impenetrable Forest shedding mountain gorilla lymphocryptovirus 1 (Gbb-LCV1) between November 2012 and June 2013 by location (Protected Area) and gorilla family group.

Conservation Region	Family Group	No. Pos / No. Tested (%)
	Agashya	11 / 25 (44%)
	Amahoro	2 / 2 (100%)
	Bwenge	1 / 6 (17%)
	Hirwa	5 / 13 (39%)
	Isabukuru	5 / 13 (39%)
	Kuryama	7 / 12 (58%)
**Virunga Volcanoes**	Kwitonda	8 / 19 (42%)
**Conservation Region**	Ntambara	4 / 9 (44%)
	Pablo	10 / 19 (53%)
	Sabinyo	4 / 9 (44%)
	Susa	17 / 22 (77%)

	Titus	5 / 9 (56%)
	Ugenda	3 / 4 (75%)
	Umubano	1 / 8 (13%)
	All Virunga Volcanoes Groups	83 / 170 (49%)
**Bwindi Impenetrable Forest**	Bitukura	2 / 15 (13%)
	Busingye	0 / 3 (0%)
	Bweza	5/13 (39%)
	Habinyanja	6 / 9 (67%)
	Kahungye	7 / 14 (50%)
	Kyagurilo	7 / 17 (41%)
	Mishaya	2 /10 (20%)
	Mubare	6 / 12 (50%)
	Nkuringo	11 / 16 (69%)
	Oruzogo	6 / 20 (30%)
	Rushegura	5 / 23 (22%)
	Shongi	1 / 5 (20%)
	All Bwindi Groups	60 / 162 (37%)
